# Reduction in Pediatric Surgery’s Emergency Department Visits During COVID-19 Pandemic in a Tertiary University General Hospital in Greece

**DOI:** 10.7759/cureus.17543

**Published:** 2021-08-29

**Authors:** Katerina Kambouri, Konstantinos Skarentzos, Panagoula Oikonomou, Eirini Papachristou, Maria Aggelidou

**Affiliations:** 1 Pediatric Surgery, Democritus University of Thrace/ General University Hospital of Alexandroupolis, Alexandroupolis, GRC; 2 Medicine, Democritus University of Thrace/ General University Hospital of Alexandroupolis, Alexandroupolis, GRC; 3 Experimental Surgery, Democritus University of Thrace/ General University Hospital of Alexandroupolis, Alexandroupolis, GRC; 4 Pediatric Surgery, General University Hospital of Alexandroupolis, Alexandroupolis, GRC

**Keywords:** coronavirus, covid-19, emergency department, pediatric surgery, greece

## Abstract

Background

From January 2020, coronavirus has caused more than three million deaths. Lockdown has been enforced in many countries worldwide, affecting the emergency department visits of many surgical specialties.

Methods

The purpose of this study was to present the difference in trends in pediatric emergency department visits from March 1 to May 30, 2020, compared to the same 3-month period in 2019 in a tertiary university hospital in Greece, which was one of the referral centers for COVID-19 patients.

Results

A 42.5% reduction in emergency department visits was observed. In 2020, only 196 patients visited the pediatric surgery emergency department, versus 341 patients in 2019 (p<0.05). The reasons for visiting the emergency department did not change in most categories. Even though visits to the emergency department were reduced, the rates of the distance of the patient’s residency from the hospital remained roughly the same. Hospital admission rates remained roughly the same.

Conclusion

Even though there was a huge decrease in numbers, the reasons for visiting the ED remained roughly the same. The only exception was indoor accidents, which increased in 2020.

## Introduction

From January to May 2020, coronavirus caused 3,206,339 deaths in the world. In Greece, 346,422 confirmed cases and 10,453 deaths have been reported. Globally, more than 563 million people have been vaccinated [1]. The pandemic has caused many changes in medicine and medical care worldwide.

In Greece, to prevent the spread of COVID-19, a strict lockdown was enforced between March and May 2020, which may affect the pathway to healthcare services [2]. During this period, hospitals canceled all elective surgical operations and prepared intensive care units (ICUs) for the expected arrival of patients with COVID-19. Moreover, people may have avoided seeking medical assistance for other diseases in hospitals because of the fear of contracting COVID-19. While some studies have referred to a reduction of adult visits to the emergency department (ED) unit, there are only sporadic studies on the behavior of the pediatric population [2-8].

The purpose of this study was to contrast trends in pediatric emergency department visits from March first to May 30, 2020, compared to the same 3-month period in 2019 in a tertiary university hospital in Greece, which was one of the referral centers for COVID-19 patients.

## Materials and methods

This retrospective observational study used data from the University General Hospital of Alexandroupolis, Greece. Our general hospital serves a large area of Greece from the Evros River to Eastern Macedonia. During the pandemic, our hospital received COVID cases from these areas. The procedures followed were in accordance with the ethical standards of the responsible committee on human experimentation (institutional or regional) and with the Helsinki Declaration of 1964, as revised in 2000 [9]. The original protocol was approved by the Medical Ethics Committee of the General Universal Hospital of Alexandroupolis, Greece. Greece faced the first lockdown during the COVID-19 pandemic from March first to May 30, 2020. This study was designed to examine the trends in pediatric emergency department visits between 2020 and 2019, when there was no lockdown or any pandemic outburst.

From March first to May 30, 2020, and from March first to May 30. In 2019, we examined the trends in pediatric surgery emergency department visits. The total number of visits was then determined. We also reported the patients’ demographics and reasons for each visit. Furthermore, the distance from the patient’s residence to the hospital was also noted. Moreover, we reported on any hospital admission. We then compared the results for 2019 and 2020. The source of the information was the entire pediatric surgery’s ER visit clinical records, which were in handwritten form. The data were abstracted from two independent authors blinded to each other's records. Then, the two records were compared to each other and a third author resolved any differences.

Regarding the etiology of emergency department visits, we separated the reasons into 11 categories. The first category was outdoor falls or accidents, which consisted of car accidents, bicycle accidents, falls, or sports injuries, resulting in head injury, thoracic, abdominal, back, or pelvic trauma. The second category was indoor fall or accident, which included falls or accidents inside the house resulting in head injury, thoracic, abdominal, back, or pelvic trauma. The third category was abdominal pain, which involved non-traumatic abdominal pain. Skin lacerations included any superficial trauma requiring stitches. Scrotum disorders mainly consisted of non-traumatic scrotum pain or scrotum hernias. Inflammation and abscesses included superficial inflamed wounds, abscesses, and skin lesions. Extremity injury was defined as any injury outdoor/indoors that affected only the legs and/or hands. Hernia was referred to as an abdominal hernia. Foreign body ingestion was ranked tenth. Last but not least, any other reason for emergency department visits that did not fit in the above 10 categories were included in the last category “other.” 

All analyses were performed using IBM SPSS statistics 25. The chi-squared test (X2) was used to analyze the data and Fisher's exact test (2-sided) values were reported. The p-value between the number of patients in the 2019 group and in the 2020 group was calculated via student t-test. The p-value between mean age in the 2019 group and in the 2020 group was calculated via independent t-test. Statistical significance was set at p < 0.05. 

## Results

In 2020, only 196 patients visited the pediatric surgery emergency department versus 341 patients in 2019 (p<0.05). Specifically, a 42.5% reduction is observed. In both periods, male patients visited the emergency department more frequently than female patients. The mean age of visitors remained roughly the same.

The reasons for visiting the emergency department did not change in 10 out of 11 categories, but indoor accidents increased in 2020 versus 2019 (p<0.05). Even though visits to the emergency department were reduced, the rates of the distance of the patient’s residence from the hospital remained roughly the same. The hospital admission rates remained roughly the same. The results are presented in Table 1.

**Table 1:** Demographics, etiology, distance from hospital, administration

Table 1: Demographics, etiology, distance from hospital, administration			
	2019	2020	p
Gender	female: 114 (33.4%)	female: 68 (34.7%)	
	male: 227 (66.6%)	male: 128 (65.3%)	0.777 ( for gender distribution)
	total: 341	total: 196	
Age	mean: 6.8 years	mean: 6.1 years	0.943
	range:14 days- 14 years	range: 2 months- 13.5 years	
Etiology	fall/accident (outdoor): 80 (23.5%)	Fall/accident (outdoor): 32 (16.3%)	0.060
	fall/accident (indoor): 47 (13.8%)	fall/accident (indoor): 58 (29.6%)	0.000015
	abdominal pain: 49 (14.4%)	abdominal pain: 30 (15.3%)	0.801
	skin laceration: 50 (14.7%)	skin laceration: 25 (12.8%)	0.606
	scrotum disorders: 27 (0.08%)	scrotum disorders: 7 (0.04%)	0.64
	inflammation/abscess: 20 (0.06%)	inflammation/abscess: 6 (0.03%)	0.213
	burn: 11 (0.03%)	burn: 3 (0.015%)	0.276
	extremity injury: 14 (0.04%)	extremity injury: 12 (0.06%)	0.303
	hernia: 15 (0.04%)	hernia: 6 (0.03%)	0.497
	foreign body ingestion.: 8 (0.02%)	foreign body ingestion.: 1 (0.005%)	0.165
	others: 20 (0.06%)	others: 10 (0.05%)	0.846
Distance from hospital	<60km: 234	<60km: 126	0.7
	60-79km: 54	60-79km: 32	0.99
	80-99km: 14	80-99km: 6	0.22
	100-149km: 21	100-149km: 19	0.6
	150-199km: 11	150-199km: 9	0.72
	>200km: 7	>200km: 4	0.1
Hospital admission	yes: 79 (23.2%)	yes: 54 (27.6%)	0.44
	no: 262 (76.8%)	no: 142 (72.4%)	

## Discussion

The COVID-19 pandemic caused a major lockdown for three months in Greece. In this period, from March first to May 30, 2020, we found a 42.5% reduction in visits to the pediatric surgery emergency department. Similar results have been reported in the USA in the general population [4]. In Germany, a 63.8% reduction in the pediatric emergency department was observed 4 weeks after lockdown [3]. Despite the huge difference in the number of patients who visited the emergency department, the reasons for visiting the emergency department remained the same. A possible explanation might be that these reasons for ER visits were not affected by the lockdown. For example, abdominal pain caused by appendicitis could not be affected by the lockdown. The only exception to this statement was the indoor fall/accident category. In this study, the number of accidents inside houses increased in 2020. A possible explanation for this increase might be that the lockdown has restricted people to stay at home, which is likely to impact their physical and mental health. Especially vulnerable to this were the children who couldn’t go out for playing and also their schools were closed. Thus, the increased rates of domestic accidents could be attributed to the fact that children spent more time at home, and in trying to play and run in a limited space, they were more prone to accidents. Our data were also consistent with a similar study conducted in Hong Kong, where domestic accidents presenting to the Pediatric Emergency Department increased significantly during the COVID-19 lockdown compared to the previous year [8].

However, we noticed a decrease in the total number of accidents (indoor/outdoor) that did not justify the total reduction of patients coming to our emergency department. A possible explanation might be that parents choose not to visit our emergency department due to fear of contracting COVID-19 because our hospital was dealing with COVID patients on a daily basis. Another explanation might be that parents were more cautious about their decision to visit an emergency department during the pandemic, so they preferred to visit an outpatient clinic instead if the severity or the urgency of the child’s symptoms allowed it.

Similar results have been reported in other medical specialties in other countries, such as ophthalmology in India. In this study, a decrease of 23.9% was reported during emergency visits [10]. In the USA, a study regarding general surgery operations reported a 33.5% reduction in total major cases performed in a 4-month period in 2020 compared to 2018 and 2019 [11].

Some limitations of our study need to be addressed. First, we only extracted data from the pediatric surgery emergency department. Thus, our findings may not be generalizable to other EDs. Even though our data showed a 42.5% reduction in visits in this three-month period (from March to May) between 2019 and 2020, we cannot determine whether children with serious symptoms, diseases, or accidents were left untreated during the COVID-19 pandemic.

## Conclusions

Our data illustrated a 42.5% reduction in pediatric surgery ED visits in the 3-month period during the lockdown versus the same period in 2019. Even though there was a huge decrease in numbers, the reasons for visiting the ED remained roughly the same. The only exception was indoor accidents, which increased in 2020. The reduction might have been due to the fear of hospital-acquired infections and inadequate primary health care. In addition, the possible increase in domestic injuries might have caused more serious damage to children’s health than the virus itself. The lessons learned from this pandemic wave suggested that governments should consider all information and adopt appropriate public health strategies in order to prevent or minimize any pediatric "collateral damage" caused by delayed response to emergencies during a possible next pandemic wave.
